# iTRAQ-Based Proteomic Analysis Reveals Potential Serum Biomarkers for Pediatric Non-Hodgkin’s Lymphoma

**DOI:** 10.3389/fonc.2022.848286

**Published:** 2022-03-17

**Authors:** Runhong Yu, Linna Cheng, Shiwei Yang, Yufeng Liu, Zunmin Zhu

**Affiliations:** ^1^ Henan Provincial People’s Hospital, Institute of Hematology of Henan Provincial People’s Hospital, Zhengzhou, China; ^2^ Henan Provincial People’s Hospital, Henan Key laboratory of Stem Cell Differentiation and Modification, Zhengzhou, China; ^3^ Department of Pediatrics, The First Affiliated Hospital of Zhengzhou University, Zhengzhou, China; ^4^ Department of Hematology, People’s Hospital of Zhengzhou University, Zhengzhou, China

**Keywords:** non-Hodgkin’s lymphoma, proteomics, children, isobaric tags for relative and absolute quantification (iTRAQ), serum

## Abstract

Non-Hodgkin’s lymphoma (NHL) is the third most common malignant tumor among children. However, at initial NHL diagnosis, most cases are at an advanced stage because of nonspecific clinical manifestations and currently limited diagnostic methods. This study aimed to screen and verify potential serum biomarkers of pediatric NHL using isobaric tags for relative and absolute quantification (iTRAQ)-based proteomic analysis. Serum protein expression profiles from children with B-NHL (n=20) and T-NHL (n=20) and healthy controls (n=20) were detected by utilizing iTRAQ in combination with two-dimensional liquid chromatography-tandem mass spectrometry (2D LC–MS/MS) and analyzed by applying Ingenuity Pathway Analysis (IPA). The candidate biomarkers S100A8 and LRG1 were further validated by using enzyme-linked immunosorbent assays (ELISAs). Receiver operating characteristic (ROC) analysis based on ELISA data was used to evaluate diagnostic efficacy. In total, 534 proteins were identified twice using iTRAQ combined with 2D LC–MS/MS. Further analysis identified 79 and 73 differentially expressed proteins in B-NHL and T-NHL serum, respectively, compared with control serum according to our defined criteria; 34 proteins were overexpressed and 45 proteins underexpressed in B-NHL, whereas 45 proteins were overexpressed and 28 proteins underexpressed in T-NHL (*p* < 0.05). IPA demonstrated a variety of signaling pathways, including acute phase response signaling and liver X receptor/retinoid X receptor (LXR/RXR) activation, to be strongly associated with pediatric NHL. S100A8 and LRG1 were elevated in NHL patients compared to normal controls according to ELISA (*p* < 0.05), which was consistent with iTRAQ results. The areas under the ROC curves of S100A8, LRG1, and the combination of S100A8 and LRG1 were 0.873, 0.898 and 0.970, respectively. Our findings indicate that analysis of the serum proteome using iTRAQ combined with 2D LC–MS/MS is a feasible approach for biomarker discovery. Serum S100A8 and LRG1 are promising candidate biomarkers for pediatric NHL, and these differential proteins illustrate a novel pathogenesis and may be clinically helpful for NHL diagnosis in the future.

## Introduction

Non-Hodgkin’s lymphoma (NHL) is the most common hematologic malignancy in the world, with numerous biologically and clinically heterogeneous subtypes (1). NHL is characterized by great differences in clinical manifestations, biological characteristics and survival outcomes among different ages groups (2). For example, pediatric NHL exhibits significant differences in the distribution of histologic subtypes compared to NHL in adults. In the pediatric age range, NHL is the third most common malignancy, accounting for approximately 6% of new cancers, and is primarily represented by only a few histologic subtypes (1, 2). Children often develop highly aggressive lymphoma. The principal subtypes by incidence in children are the mature B-cell lymphomas Burkitt lymphoma/leukemia (BL), lymphoblastic lymphomas (LBL) of the precursor B- or T-cell type and diffuse large B-cell lymphomas (3). Compared to Hodgkin’s lymphomas (HLs), NHLs are more common in childhood and have considerable phenotypic and biological heterogeneity. Pediatric NHL usually presents as aggressive disseminated disease or as a rapidly growing mass with multicenter origin, long-distance transmission, and extranodal infiltration, as well as great differences in clinical manifestations. Therefore, most NHLs are diagnosed as stage III or IV (4). At present, there is no specific early diagnostic method or standard treatment for NHL in children. Without timely diagnosis and reasonable treatment, children with NHL may die in a short time and have an overall poor prognosis (4). The initial manifestations of some pediatric NHLs are complex, diverse and lack specificity. In addition, the poor expression ability of children and the concealment of some disease sites often lead to missed diagnosis or misdiagnosis (5).

The incidence of NHL is increasing yearly, and the clinical prognosis of early and late cases is very different. Consequently, it is of great significance to explore early diagnosis indicators for NHL prognosis. Serum tumor markers are commonly used as clinical reference indicators for tumor diagnosis, with an important role in the diagnosis of various malignant tumors. Thus, the screening of new biomarkers for the early diagnosis of pediatric NHL, as well as potential drug targets, is urgently needed. Furthermore, it has important clinical value for the in-depth study of the pathogenesis of NHL and for the identification of new therapeutic targets.

Proteomic-based approaches have become a very powerful technique for biomarker screening (6, 7). Among them, isobaric tags for relative and absolute quantification (iTRAQ) is extensively utilized as a high-content screening assay for identifying cancer protein biomarkers and is able to analyze eight kinds of different samples at the same time (8–10). In our research, we applied an iTRAQ-based approach to detect the differences in serum proteomes from NHL patients and controls (B-NHL *vs*. control and T-NHL *vs*. control), which was followed by bioinformatic analysis utilizing Ingenuity Pathway Analysis (IPA) to evaluate the changed proteome associated with NHL in children. Our findings will help identify potential serum biomarkers of pediatric NHL and explore their clinical value.

## Materials and Methods

### Clinical Serum Sample Collection

All serum specimens of children with B-NHL and T-NHL and healthy controls were collected from the First Affiliated Hospital of Zhengzhou University. This study followed the Declaration of Helsinki and was approved by the Institutional Ethics Committee of the Department of Medicine of the First Affiliated Hospital of Zhengzhou University. Informed consent was obtained from the parents or guardians prior to study initiation. All serum samples were discarded after clinical use. Newly diagnosed patients with NHL without radiotherapy or chemotherapy between January 2013 and December 2014 were included in this analysis. The diagnosis of primary NHL was based on a histological and immunohistochemical examination of tumor tissue and was classified according to the 2016 World Health Organization criteria (3). The staging of NHL was based on the St. Jude classification by Murphy, which has been used since 1980 (11). The essential features of the research subjects are shown in **Table 1**. Serum samples were stored at −80°C until analysis.

**Table 1:** Essential features of the research subjects.

	B-NHL	T-NHL	Control
No. of cases	20	20	44
Gender			
Male	15	17	22
Female	5	3	22
Median age, years (range)	7(1.2-14)year	9(0.9-13)year	7(1-14)year
St. Jude stage			
I		0	–
II	2	0	–
III	2	8	–
IV	16	12	–

### Sample Preparation and iTRAQ Labeling

The serum in the same volume from each of the 20 subjects in each group (B-NHL, T-NHL and healthy controls) was mixed. Then, the high-abundance proteins of each group were removed by applying the Human 14 Multiple Affinity Removal System (Agilent, Santa Clara, CA, USA). Proteins were extracted, and the concentration was measured using a Bradford Protein Assay Kit (Bio-Rad, Richmond, CA). For each sample, 100 μg protein (Control, B-NHL and T-NHL) was reductively alkylated, and trypsin was used for enzymatic hydrolysis. The resulting peptides were labeled with the 8-plex iTRAQ reagent kit according to the manufacturer’s protocol (Applied ABI, USA), and the conditions for each labeled reagent was performed as follows: Control group, iTRAQ reagent 113; B-NHL group, iTRAQ reagent 119 and T-NHL, iTRAQ reagent 121. The detailed procedures of iTRAQ have been described previously (8). After labeling, the three samples were mixed and desalted using a Sep-Pak Vac C18 column (Waters, USA) and vacuum-dried.

### 2D LC-MS/MS Analysis and Data Analysis

Peptides were fractioned, identified and quantified as previously described (8). In brief, labeled peptides were pre-separated using an ultraperformance liquid chromatography (UPLC) system with a C18 reverse-phase BEH column (50×2.1 mm, 1.7 µm, 130 Å, Waters, USA). The fractions were separated using a nano-HPLC system with a secondary reversed-phase analytical column (C18 column, 150 mm ×75 µm, 3 µm, 300 Å, Eksigent, USA) system and then subjected to ESI-Q-TOF mass spectrometry (Triple TOF4600, AB SCIEX, USA) for protein identification. For MS scans, the m/z scan range was 350–1250 Da. For MS/MS scans, the m/z scan range was 100–1250. Mascot 2.3.02 (Matrix Science, London, UK) and Scaffold (Proteome Software, Portland, OR, USA) software were used for the identification and quantification of proteins based on data from the international Swiss-Prot human database (20111015, human). Only proteins identified with at least 95% confidence, a fold change>1.2 (ratio > 1.2 or < 0.8) and a *p* value<0.05 were considered to be differentially expressed proteins (DEPs).

### Bioinformatic Analyses

Ingenuity Pathway Analysis software (IPA) (version 7.1, Ingenuity System Inc., Redwood City, CA, USA; www.ingenuity.com) was used to analyze the biological functions, predominant canonical pathways, and protein-interaction networks associated with the DEPs. Significance levels were assessed by Fisher’s exact tests (*p*<0.05).

### Enzyme-Linked Immunosorbent Assay-Based Validation

Serum concentrations of S100A8 and LRG1 were measured using human S100A8 and LRG1 ELISA assay kits (Uscn, China), respectively. ELISAs were performed as previously described (12) according to manufacturer’s instructions. The absorbance values of the standards and samples were determined by spectrophotometry at 450 nm using a microplate reader. Each sample was analyzed in duplicate.

### Statistical Analysis

All data are presented as the mean ± standard deviation, with one-way analysis of variance (ANOVA) and unpaired Student’s t-tests employed for comparisons among the three experimental groups. The statistical analyses were performed using IBM SPSS Statistics for Windows (version 20.0, Armonk, NY, USA). Receiver operating characteristic (ROC) curves and the area under the ROC curve (AUC) were used to access the sensitivity and specificity, with *p <*0.05 considered statistically significant.

## Results

**Figure 1:**
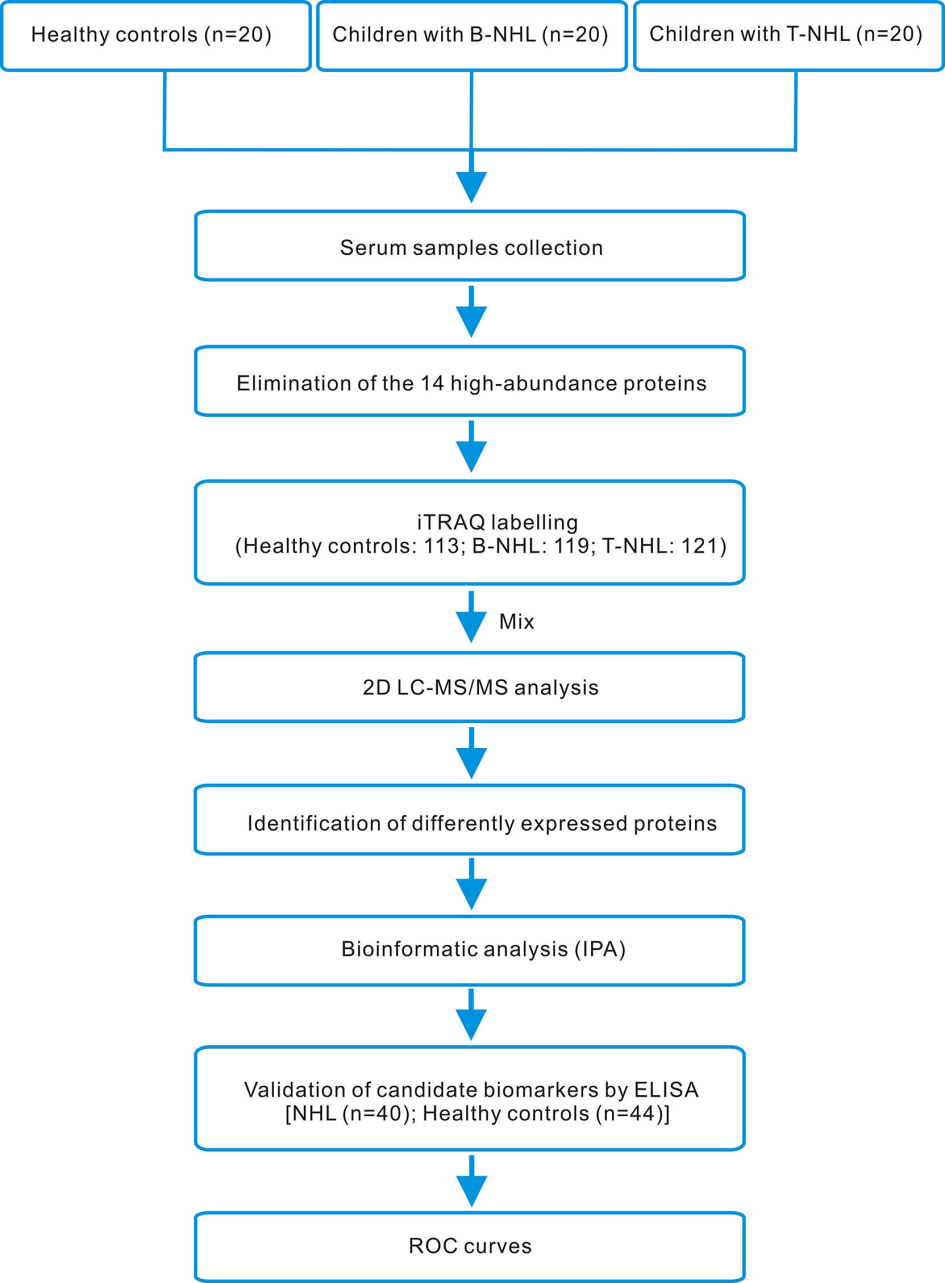
iTRAQ-based proteomics workflow of this study. Serum samples from healthy controls and children with B-NHL and T-NHL were pooled. After elimination of the 14 high-abundance proteins by applying the Human 14 Multiple Affinity Removal System (MARS), tryptic digestion and iTRAQ labeling, the peptide mixture was analyzed by 2D LC–MS/MS. The differentially expressed proteins were analyzed by the bioinformatics software IPA. The candidate biomarkers S100A8 and LRG1 were selected for further validation using ELISA. The efficacy of the candidate biomarkers was then evaluated using ROC curves.

In our research, we utilized iTRAQ combined with two-dimensional liquid chromatography-tandem mass spectrometry (2D LC–MS/MS) to screen serum DEPs, followed by enrichment analysis using IPA software and clinical validation by ELISA. The whole workflow of this study is shown in **Figure 1**.

### Profiling of Serum Proteomes With iTRAQ-Based Proteomic Analysis

**Figure 2:**
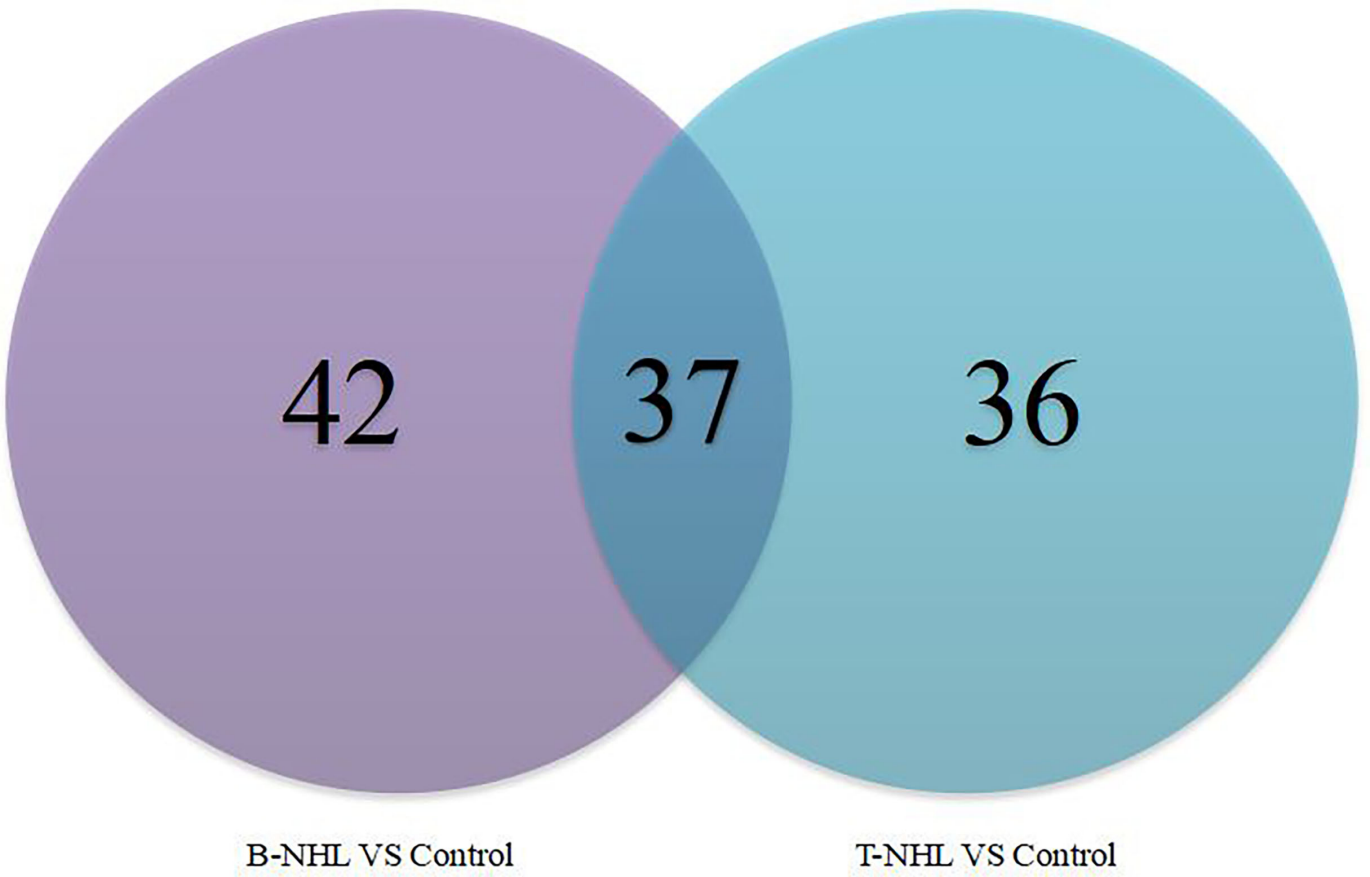
Venn diagram indicating the serum differentially expressed proteins between the pediatric B-NHL and T-NHL groups and the control group.

To identify novel serum biomarkers of pediatric B-NHL and T-NHL, the proteomes of serum samples from children with B-NHL (n=20) and T-NHL (n=20) and healthy controls (n=20) were quantitatively profiled using iTRAQ combined with 2D LC–MS/MS. As a result, we identified 534 nonredundant proteins containing at least one peptide. Detailed information on the identified proteins and the raw MS data are provided in **Additional File 1**. We defined proteins with a change in relative abundance greater than 1.2 times and *p <*0.05 as significant. Based on the cutoff value, the iTRAQ-based analyses revealed that the levels of 34 and 45 proteins were elevated and decreased, respectively, in B-NHL compared with healthy controls. Moreover, 45 and 28 proteins were overexpressed and underexpressed, respectively, in T-NHL compared with healthy controls. The results suggested that these DEPs had the potential to be relevant markers for pediatric NHL screening. The details of all DEPs are shown in **Table 2** and **Figure 2**. Further analysis showed that the quantity of overexpressed proteins was greater than that of underexpressed proteins in the T-NHL group compared with the control group. In comparing the B-NHL group with the control group, the numbers of upregulated and downregulated proteins were similar. Moreover, among the DEPs, 19 overexpressed and 17 underexpressed proteins were shared by both B-NHL and T-NHL. 42 and 36 DEPs were specifically implicated in B-NHL and T-NHL, respectively. In addition, thymosin beta-4 was overexpressed in T-NHL but was underexpressed in B-NHL.

**Table 2:** Differentially expressed proteins identified in B-NHL and T-NHL compared with the control by iTRAQ-based proteomic analysis.

Accession Number	Protein Name	Gene Name	Average Ratio	
			B-NHL VS Control	T-NHL VS Control
CFAB_HUMAN	Complement factor B	CFB	1.22	–
CO3_HUMAN	Complement C3	C3	2.32	2
APOA4_HUMAN	Apolipoprotein A-IV	APOA4	0.55	0.55
THRB_HUMAN	Prothrombin	F2	0.78	–
ITIH3_HUMAN	Inter-alpha-trypsin inhibitor heavy chain H3	ITIH3	1.39	1.22
CO9_HUMAN	Complement component C9	C9	1.75	–
LUM_HUMAN	Lumican	LUM	0.5	0.44
CXCL7_HUMAN	Platelet basic protein	PPBP	0.73	–
A2GL_HUMAN	Leucine-rich alpha-2-glycoprotein 1	LRG1	1.5	2
PLSL_HUMAN	Plastin-2	LCP1	2.63	–
FIBA_HUMAN	Fibrinogen alpha chain	FGA	0.58	0.63
APOA2_HUMAN	Apolipoprotein A-II	APOA2	3.35	4.24
IC1_HUMAN	Plasma protease C1 inhibitor	SERPING1	2.17	2
ACTG_HUMAN	Actin, cytoplasmic 2	ACTG1	1.26	1.26
A2AP_HUMAN	Alpha-2-antiplasmin	SERPINF2	0.75	–
TETN_HUMAN	Tetranectin	CLEC3B	0.62	0.67
PGRP2_HUMAN	N-acetylmuramoyl-L-alanine amidase	PGLYRP2	0.7	0.7
TSP1_HUMAN	Thrombospondin-1	THBS1	0.7	–
SHBG_HUMAN	Sex hormone-binding globulin	SHBG	0.65	0.6
APOC3_HUMAN	Apolipoprotein C-III	APOC3	1.62	1.48
PERM_HUMAN	Myeloperoxidase	MPO	1.94	2.35
FA12_HUMAN	Coagulation factor XII	F12	0.55	–
PPIA_HUMAN	Peptidyl-prolyl cis-trans isomerase A	PPIA	1.3	1.55
CADH5_HUMAN	Cadherin-5	CDH5	0.7	0.75
TENX_HUMAN	Tenascin-X	TNXB	0.72	–
APOC2_HUMAN	Apolipoprotein C-II	APOC2	1.55	–
CYTC_HUMAN	Cystatin-C	CST3	0.75	–
SEPP1_HUMAN	Selenoprotein P	SEPP1	0.65	–
FIBB_HUMAN	Fibrinogen beta chain	FGB	0.42	0.58
CHLE_HUMAN	Cholinesterase	BCHE	0.62	0.67
SAA1_HUMAN	Serum amyloid A-1 protein	SAA1	2	–
SAMP_HUMAN	Serum amyloid P-component	APCS	1.6	1.6
HGFL_HUMAN	Hepatocyte growth factor-like protein	MST1	0.75	–
APOC1_HUMAN	Apolipoprotein C-I	APOC1	1.45	1.25
COMP_HUMAN	Cartilage oligomeric matrix protein	COMP	0.45	0.45
CO4A_HUMAN	Complement C4-A	C4A	1.25	–
PLF4_HUMAN	Platelet factor 4	PF4	0.5	0.5
PI16_HUMAN	Peptidase inhibitor 16	PI16	0.23	0.23
S10A8_HUMAN	Protein S100-A8	S100A8	4.53	5.95
FBLN1_HUMAN	Fibulin-1	FBLN1	0.73	–
HABP2_HUMAN	Hyaluronan-binding protein 2	HABP2	0.7	–
PGBM_HUMAN	Basement membrane-specific heparan sulfate proteoglycan core protein	HSPG2	0.7	–
FA11_HUMAN	Coagulation factor XI	F11	0.6	–
1433Z_HUMAN	14-3-3 protein zeta/delta	YWHAZ	4	3.75
FHR1_HUMAN	Complement factor H-related protein 1	CFHR1	1.5	–
VIME_HUMAN	Vimentin	VIM	2.17	–
TYB4_HUMAN	Thymosin beta-4	TMSB4X	0.75	1.25
FHR5_HUMAN	Complement factor H-related protein 5	CFHR5	1.23	–
GP1BA_HUMAN	Platelet glycoprotein Ib alpha chain	GP1BA	0.65	–
ATRN_HUMAN	Attractin	ATRN	0.68	–
SODE_HUMAN	Extracellular superoxide dismutase [Cu-Zn]	SOD3	0.68	–
CO1A1_HUMAN	Collagen alpha-1(I) chain	COL1A1	0.18	0.18
CD166_HUMAN	CD166 antigen	ALCAM	0.65	–
LDHA_HUMAN	L-lactate dehydrogenase A chain	LDHA	2.72	–
CHL1_HUMAN	Neural cell adhesion molecule L1-like protein	CHL1	0.75	–
SAA4_HUMAN	Serum amyloid A-4 protein	SAA4	1.82	2.36
PTGDS_HUMAN	Prostaglandin-H2 D-isomerase	PTGDS	0.6	0.45
COBA2_HUMAN	Collagen alpha-2(XI) chain	COL11A2	0.5	–
TRML1_HUMAN	Trem-like transcript 1 protein	TREML1	1.25	1.25
F13A_HUMAN	Coagulation factor XIII A chain	F13A1	0.73	–
FIBG_HUMAN	Fibrinogen gamma chain	FGG	0.61	0.67
COTL1_HUMAN	Coactosin-like protein	COTL1	1.45	–
C4BPA_HUMAN	C4b-binding protein alpha chain	C4BPA	1.31	–
ICAM2_HUMAN	Intercellular adhesion molecule 2	ICAM2	0.68	–
BASP1_HUMAN	Brain acid soluble protein 1	BASP1	1.8	–
ANAG_HUMAN	Alpha-N-acetylglucosaminidase	NAGLU	0.75	–
PEBP1_HUMAN	Phosphatidylethanolamine-binding protein 1	PEBP1	1.75	1.6
COL11_HUMAN	Collectin-11	COLEC11	0.78	0.72
TARSH_HUMAN	Target of Nesh-SH3	ABI3BP	0.67	–
ICOSL_HUMAN	ICOS ligand	ICOSLG	0.6	–
G6PE_HUMAN	GDH/6PGL endoplasmic bifunctional protein	H6PD	1.25	–
TTHY_HUMAN	Transthyretin	TTR	0.35	0.5
SRGN_HUMAN	Serglycin	SRGN	0.65	–
LYVE1_HUMAN	Lymphatic vessel endothelial hyaluronic acid receptor 1	LYVE1	0.7	–
TSP4_HUMAN	Thrombospondin-4	THBS4	0.5	–
PSA1_HUMAN	Proteasome subunit alpha type-1	PSMA1	2.4	1.3
SH3L1_HUMAN	SH3 domain-binding glutamic acid-rich-like protein	SH3BGRL	1.75	2.1
FKB1A_HUMAN	Peptidyl-prolyl cis-trans isomerase FKBP1A	FKBP1A	1.45	1.45
IBP6_HUMAN	Insulin-like growth factor-binding protein 6	IGFBP6	1.33	–
AACT_HUMAN	Alpha-1-antichymotrypsin	SERPINA3	–	1.45
ITIH4_HUMAN	Inter-alpha-trypsin inhibitor heavy chain H4	ITIH4	–	2
FINC_HUMAN	Fibronectin	FN1	–	2.67
ZA2G_HUMAN	Zinc-alpha-2-glycoprotein	AZGP1	–	1.64
ANGT_HUMAN	Angiotensinogen	AGT	–	1.33
HRG_HUMAN	Histidine-rich glycoprotein	HRG	–	0.72
AMBP_HUMAN	Protein AMBP	AMBP	–	1.35
VWF_HUMAN	von Willebrand factor	VWF	–	2.21
THBG_HUMAN	Thyroxine-binding globulin	SERPINA7	–	0.7
TPM4_HUMAN	Tropomyosin alpha-4 chain	TPM4	–	1.23
MOES_HUMAN	Moesin	MSN	–	1.75
PON1_HUMAN	Serum paraoxonase/arylesterase 1	PON1	–	2.88
PROF1_HUMAN	Profilin-1	PFN1	–	1.47
VINC_HUMAN	Vinculin	VCL	–	1.31
ACTN1_HUMAN	Alpha-actinin-1	ACTN1	–	11.07
TRY1_HUMAN	Trypsin-1	PRSS1	–	1.25
CFAD_HUMAN	Complement factor D	CFD	–	1.41
UBR1_HUMAN	E3 ubiquitin-protein ligase UBR1	UBR1	–	1.9
POSTN_HUMAN	Periostin	POSTN	–	0.65
MBL2_HUMAN	Mannose-binding protein C	MBL2	–	0.55
SPRL1_HUMAN	SPARC-like protein 1	SPARCL1	–	1.45
SRCRM_HUMAN	Scavenger receptor cysteine-rich domain-containing protein	SCART1	–	0.7
APOM_HUMAN	Apolipoprotein M	APOM	–	1.35
COF1_HUMAN	Cofilin-1	CFL1	–	1.25
COR1A_HUMAN	Coronin-1A	CORO1A	–	4.42
KIT_HUMAN	Mast/stem cell growth factor receptor Kit	KIT	–	0.5
1433G_HUMAN	14-3-3 protein gamma	YWHAG	–	2.65
CAH2_HUMAN	Carbonic anhydrase 2	CA2	–	1.84
B2MG_HUMAN	Beta-2-microglobulin	B2M	–	0.45
PCOC1_HUMAN	Procollagen C-endopeptidase enhancer 1	PCOLCE	–	0.75
GGH_HUMAN	Gamma-glutamyl hydrolase	GGH	–	0.7
EGLN_HUMAN	Endoglin	ENG	–	0.75
1B07_HUMAN	HLA class I histocompatibility antigen,B-7 alpha chain	HLA-B	–	1.25
CNTN1_HUMAN	Contactin-1	CNTN1	–	0.75
MTG2_HUMAN	Mitochondrial ribosome-associated GTPase 2	MTG2	–	1.45
GGCT_HUMAN	Gamma-glutamylcyclotransferase	GGCT	–	26.78

### Bioinformatics Analysis of DEPs

**Figure 3:**
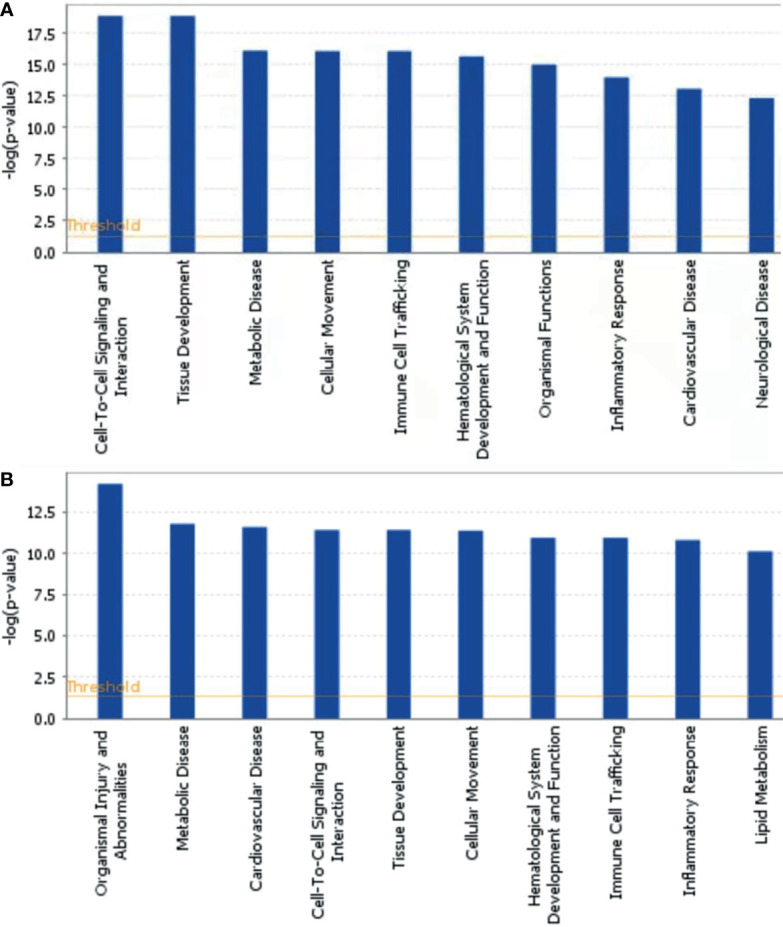
Predominant molecular functions based on differentially expressed proteins in B-NHL **(A)** and T-NHL **(B)** compared with the control. The cutoff of *p* value was set to 0.05.

**Figure 4:**
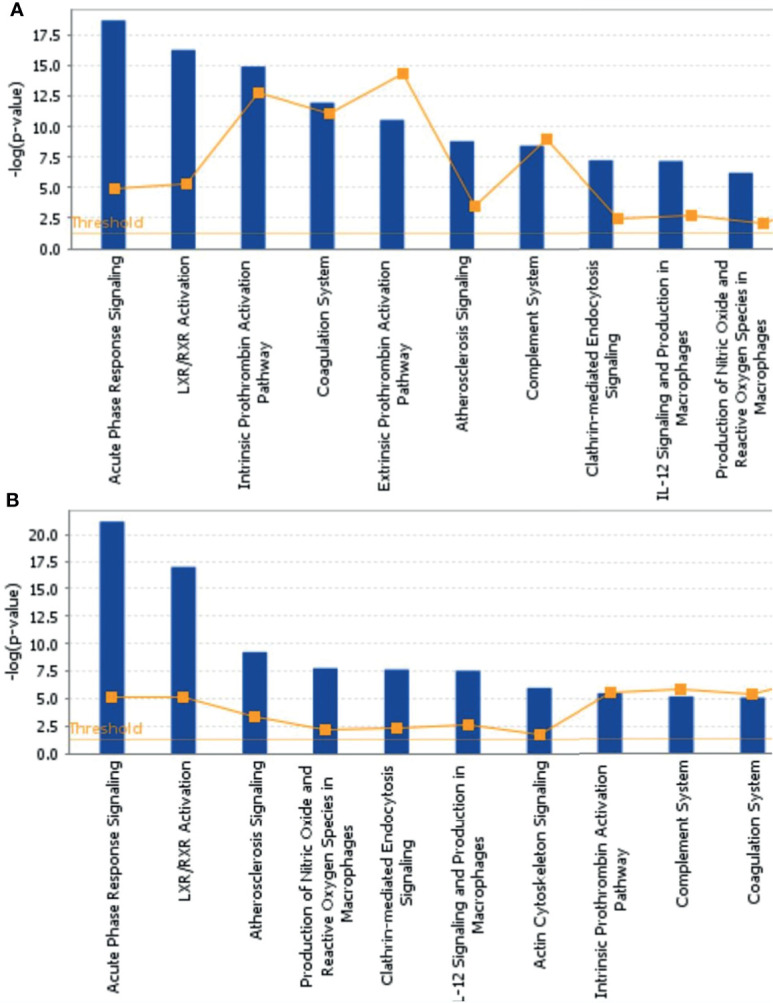
Predominant canonical pathways based on differentially expressed proteins in B-NHL **(A)** and T-NHL **(B)** compared with the control. The cutoff of *p* value was set to 0.05.

To further explore the biological functions, pathways, and networks likely involved in pediatric NHL, we subjected the DEPs to IPA. The top 10 significant biological functions and canonical pathways are listed in **Figures 3**, **4**, respectively. Among them, the functional analysis suggested that similar results were shared by the B-NHL and T-NHL groups. In IPA canonical pathway analysis, acute phase response signaling and liver X receptor/retinoid X receptor (LXR/RXR) activation were the 2 most prominent enriched pathways. The enriched pathways of DEPs in the B-NHL group mostly overlapped with those in the T-NHL group in comparison with the control group.

**Figure 5:**
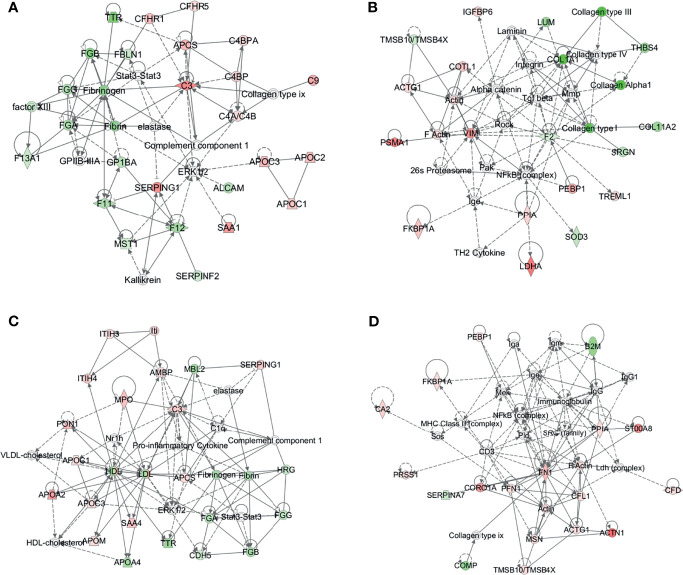
Protein–protein interaction (PPI) networks based on the differentially expressed proteins by IPA. The 2 representative PPI networks based on the differentially expressed proteins in B-NHL compared with the control **(A, B)**. The 2 representative PPI networks based on the differentially expressed proteins in T-NHL compared with the control **(C, D)**. Red denotes upregulated proteins, while green denotes downregulated proteins. Solid and dashed lines represent direct and indirect interactions, respectively.

To further understand the DEPs in the B-NHL and T-NHL groups, protein–protein interaction network analyses were carried out. The results were shown in **Additional Files 2** **,** **3**. In B-NHL, the 2 most significant networks were involved in hematological system development and function/organismal functions/humoral immune response, including 12 overexpressed and 12 underexpressed target molecules (score = 47), and cancer/gastrointestinal disease/cellular assembly and organization, with a score of 36 and containing 10 overexpressed and 8 underexpressed proteins (**Figures 5A, B**). The top 2 prominent networks in T-NHL were closely related to lipid metabolism/molecular transport/small molecule biochemistry, containing 13 upregulated and 8 downregulated proteins (score = 43), and dermatological diseases and conditions/immunological disease/inflammatory disease, with a score of 39 and containing 15 overexpressed and 3 underexpressed proteins (**Figures 5C, D**).

### Verification of DEPs by ELISA

**Figure 6:**
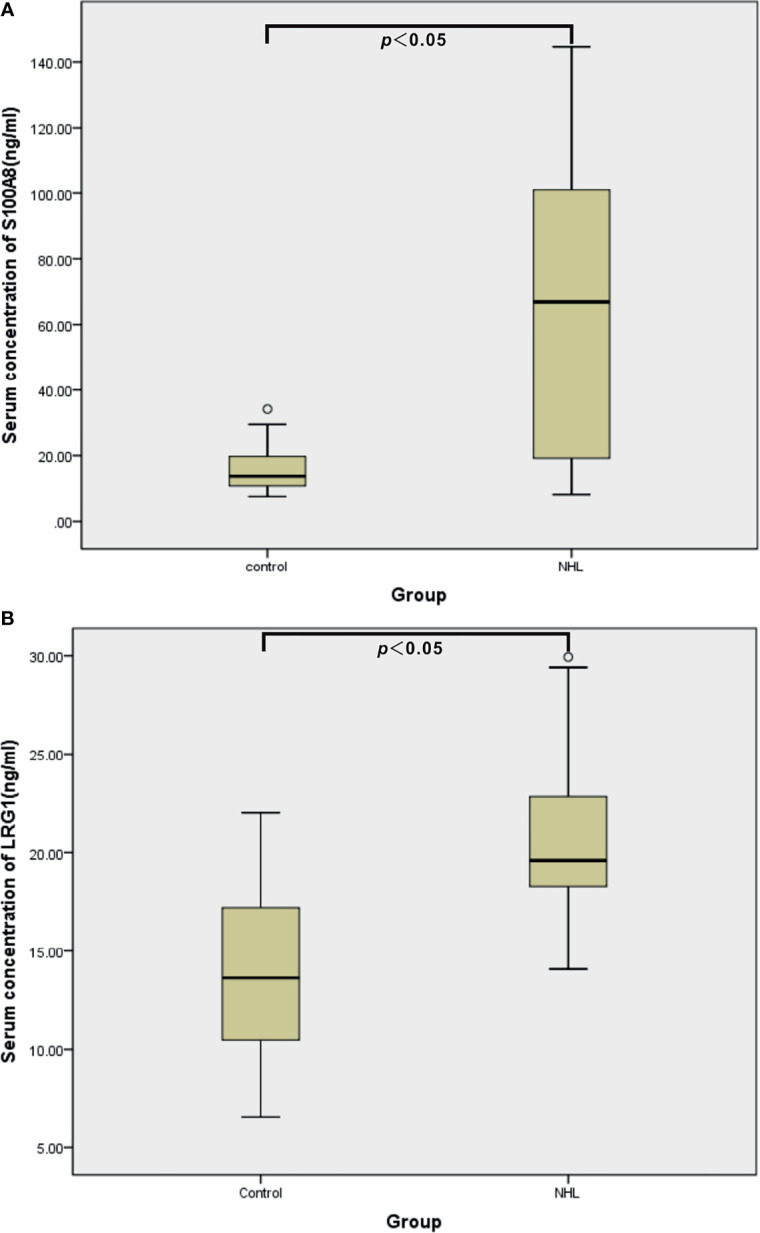
ELISA validation of serum S100A8 **(A)** and LRG1 **(B)** in healthy controls (n = 44) and children with NHL (n = 40).

To ascertain the clinical relevance of the iTRAQ results, the DEPs S100A8 and LRG1 were selected for further validation with sandwich ELISA in the individual serum specimens of 44 controls, 20 B-NHL patients and 20 T-NHL patients. The mean, SD and distribution of the protein concentrations in each group are shown in **Table 3** and **Figure 6**. The experimental results demonstrated that the serum expression levels of S100A8 and LRG1 were significantly elevated in pediatric NHL compared to healthy children (*p* < 0.05).

**Table 3:** Serum concentrations of S100A8 and LRG1 in pediatric NHL patients and healthy controls.

	NHL	Control	*p* value
S100A8 (ng/ml)	65.63 ± 39.86	15.24 ± 6.88	< 0.05
LRG1 (ng/ml)	20.94 ± 4.00	14.00 ± 3.84	< 0.05

### Areas Under the Receiver Operating Characteristic (ROC) Curves (AUCs)

**Figure 7:**
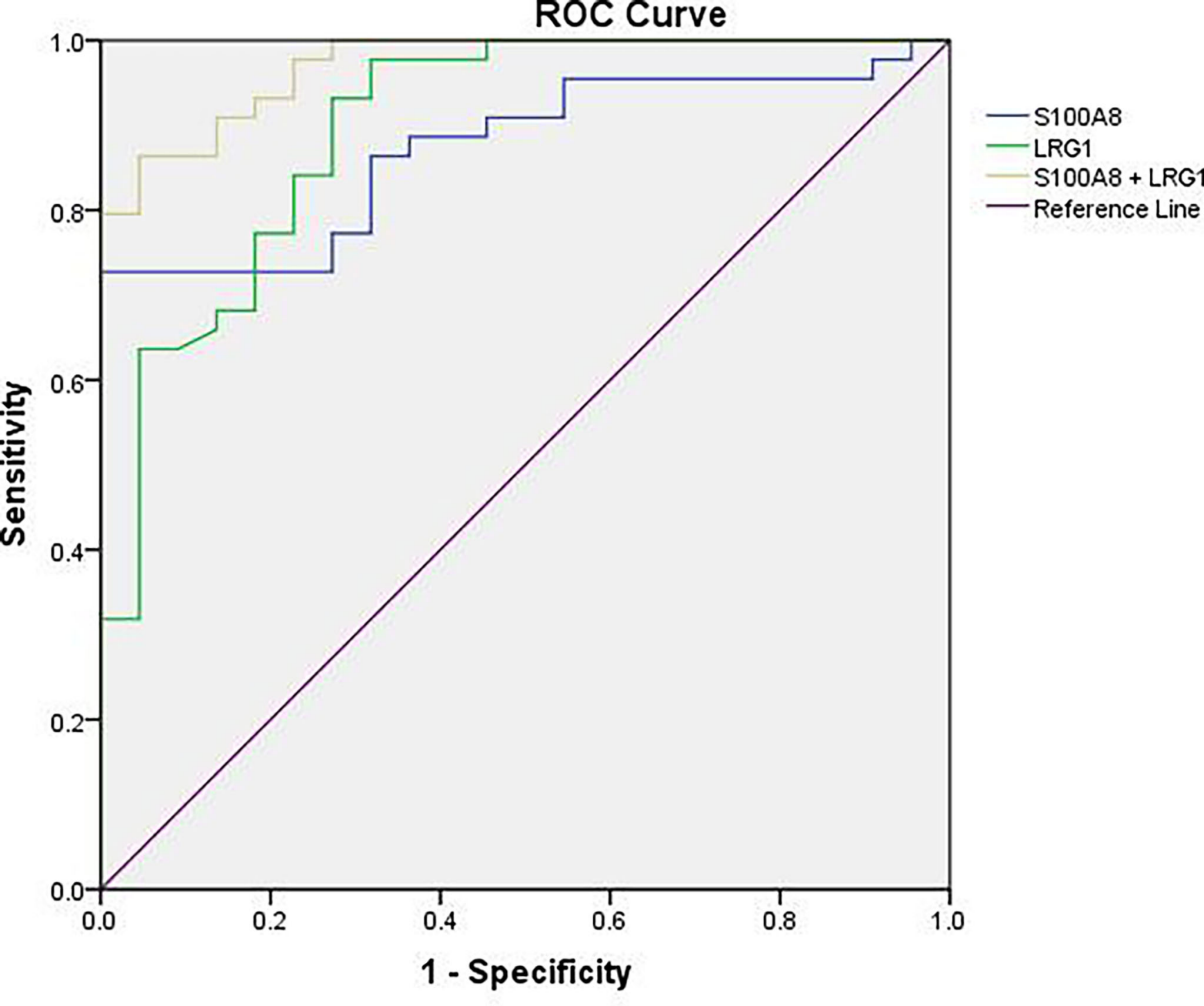
ROC curve analysis for the sensitivity and specificity of S100A8 alone, LRG1 alone, and their combination in the diagnosis of NHL.

To further estimate the efficacy of serum S100A8 and LRG1 in differentiating children with NHL from healthy controls, ROC curves were generated, as shown in **Table 4** and **Figure 7**. Our results presented unsatisfactory ROC curves of S100A8 and LRG1, which might be ascribed to the small sample size. As a result, the AUCs of S100A8, LRG1, and the combination of S100A8 and LRG1 were 0.873, 0.898 and 0.970, respectively. Serum S100A8 was able to discriminate NHL patients from healthy controls with a cutoff value of 37.880 ng/mL, with a sensitivity and specificity of 72.7% and 100%, respectively. With a cutoff value of 16.316 ng/mL, LRG1 was able to detect NHL with a sensitivity and specificity of 93.2% and 72.7%, respectively. The AUCs for differentiating between the NHL and control groups were 0.873 (95% confidence interval [CI]: 0.790-0.956) for S100A8 and 0.898 (95% CI: 0.816–0.980) for LRG1. Furthermore, the screening efficacy of S100A8 combined with LRG1 was better than that of each marker alone (95% CI: 0.937–1.000). Nevertheless, these results need clinical verification on a large scale.

**Table 4:** Receiver operating characteristics curves of the DEPs S100A8 and LRG1 in distinguishing pediatric NHL patients from healthy controls individually and in combination.

Proteins	AUC (95% CI)	*p* value	Cutoff value (ng/mL)	Sensitivity	Specificity
S100A8	0.873(0.790-0.956)	0.000	37.880	0.727	1.000
LRG1	0.898(0.816–0.980)	0.000	16.316	0.932	0.727
S100A8+ LRG1	0.970(0.937–1.000)	0.000		0.864	0.955

AUC, area under curve; CI, confidence interval.

## Discussion

Pediatric NHL is a heterogeneous group of lymphoid malignancies excluding HL. NHL is the third most common malignant tumor among children aged 1 to 14 years, second only to acute leukemia and central nervous system neoplasms (1). With the marked progress in studies and chemotherapy regimens, the prognosis of pediatric NHL has been significantly improved internationally through the adoption of effective treatment strategies according to different pathological subtypes, with an overall survival rate now exceeding 80% (2). In addition to accurate pathological diagnosis, most studies have shown that the stage of NHL has a significant impact on prognosis. Compared with adult NHL, pediatric NHL is highly invasive and is characterized by a multicenter origin, long-distance dissemination and extranodal infiltration (13). The clinical manifestations of pediatric NHL vary greatly, presenting a highly invasive process. Therefore, most children are already in stage III and IV at the time of initial diagnosis.

In our study all B-NHL cases were BL, and all T-NHL cases were T-LBL. BL is the most common pathological type of pediatric NHL, accounting for 35%~40%, with significantly more males than females being affected. BL is a highly aggressive B-cell NHL and prone to central nervous system (CNS) invasion and early bone marrow metastasis (2, 14). It is reported that the majority (80%) of T-NHL is of T-cell origin, rather than B-cell origin, in children (2). T-LBL generally presents with a mediastinal mass and advanced-stage disease and may involve the bone marrow and, less often, the CNS at diagnosis (4). In this study, most of the children were males, consistent with a previous report (15). Furthermore, Almost all cases were in stage III or IV at the time of initial diagnosis, and there were no stage I cases. It is possible that the children in our study were diagnosed late and in critical condition. In addition, many doctors in China are not well informed about NHL, and early diagnosis needs to be improved. Early diagnosis of NHL can save a number of lives and reduce the physical and economic burden of disease management at advanced stages. Nevertheless, at onset, most patients diagnosed with NHL are in an advanced stage (stages III and IV) because of nonspecific clinical manifestations and the currently limited diagnostic methods (4, 5). The discovery of serum biomarkers that are noninvasive and have high efficacy for pediatric NHL detection can greatly improve the early diagnosis of NHL. For this purpose, the serum proteome of pediatric NHL was quantitatively determined in our research.

Serum is the most easily available specimen in clinical practice. Serum quantitative proteomics is a new technology to screen serum markers of diseases developed in recent years (6, 16). In this study, we aimed to discover useful serum biomarkers of pediatric B-NHL and T-NHL. For this purpose, the serum proteomes of B-NHL, T-NHL and healthy children were globally analyzed by utilizing iTRAQ in combination with 2D LC–MS/MS. The iTRAQ analysis identified 534 proteins, among which 79 and 73 DEPs were identified in the serum of B-NHL and T-NHL patients, respectively, compared with the controls according to our defined criteria. Further analysis showed that 34 and 45 were elevated and decreased, respectively, in children with B-NHL in comparison to those in the control group. Moreover, we identified 45 overexpressed and 28 underexpressed proteins in the T-NHL *vs*. control comparison. These results indicate that there are significant differences in serum proteins between NHL patients and healthy children, which may be used as candidate markers of NHL.

Furthermore, with the DEPs in B-NHL and T-NHL, IPA analysis revealed that the biological functions of tissue development, metabolic disease and cellular movement were highlighted. These biological functions are closely related to essential tumorigenesis processes. In line with the results of functional analysis, IPA analysis showed multiple pathways to be involved in the DEPs of the B-NHL and T-NHL groups, with the most significantly influenced pathways being acute phase response signaling and the LXR/RXR activation pathway. Acute phase response signaling is a nonspecific physiological and biochemical reaction to tissue damage, infection, inflammation, and cancer. The acute phase response is a significant component part of anticancer responses and is closely related to inflammation (17). Inflammation is a recognized hallmark of cancer that contributes to the initiation and progression of a tumor (18). Moreover, there is growing evidence that inflammation plays an important role in the progression of tumors and survival of patients with cancer (18, 19). The LXR/RXR activation pathway is involved in the modulation of cholesterol metabolism, glucose metabolism, and inflammatory responses (20–22). Numerous studies have suggested that activating LXRs could restrain carcinogenesis and accelerate cancer cell apoptosis, which makes LXRs a potential tumor therapeutic target (23–26). Our results suggest that the acute phase response signaling pathway and LXR/RXR activation pathway might play a role in the tumorigenesis of pediatric NHL. Moreover, it has been reported that aberrant lipid metabolism is one of the main aspects in cancer cells and can affect tumor progression (27, 28). Apolipoprotein A-II (APOA2) was identified as a minimally invasive biomarker for detecting pancreatic cancer, lung cancer and renal cell cancer (29–31). In our research, we found that APOA2 was also overexpressed in NHL in comparison to the control, indicating that lipid metabolism might be abnormal in pediatric NHL. APOA2 may play a critical role in pediatric B-NHL and T-NHL; nevertheless, the specific role of APOA2 in NHL is not completely clear. In the future, the role of APOA2 in pediatric NHL needs to be further studied. Another upregulated protein, YWHAZ, also known as 14-3-3ζ, is commonly overexpressed in a variety of neoplasms and plays a critical role in various cancers, including breast (32), lung (33), prostate (34), hepatocellular (35), and colorectal cancers (36). Emerging evidence has indicated that YWHAZ participates in a variety of important cellular processes, such as cell proliferation, cell cycle progression, apoptosis, migration, and invasion (37).

We performed an extensive and rigorous literature analysis of DEPs in order to evaluate potential biomarkers for pediatric NHL. In this study, S100A8 and LRG1, which were identified using our iTRAQ-based proteomics method and verified using ELISA, might be promising serum markers for pediatric NHL. We found that S100A8 and LRG1 were obviously elevated in pediatric NHL compared with the control. The changes in S100A8 and LRG1 proteins could supply valuable information to diagnose pediatric NHL.

Among the upregulated DEPs, S100A8 is highly overexpressed in multifarious tumors and can promote the initiation and progression of cancers by regulating tumor cell movement, proliferation, differentiation, apoptosis and drug resistance (38–40). Aberrantly overexpressed S100A8 is also related to the metastasis and prognosis of tumors (40). Studies on hematological malignancies have found that the expression levels of S100A8 and S100A9 were markedly increased in acute myeloid leukemia (AML), and high expression of S100A8 was a poor prognostic factor for AML patients (41, 42). Furthermore, S100A8 could inhibit differentiation and maintain the AML immature phenotype (41). S100A8 often heterodimerizes with S100A9 through chemical binding, and serum S100A8/A9 may be potential biomarkers for the treatment response unrelated to inflammation in HL (43). The levels of S100A8/A9 in saliva can help differentiate the subgroups of Sjögren’s syndrome with lymphoma risk (44). A recent report found that S100A8 could promote chemoresistance to adriamycin and vincristine by enhancing autophagy in B-cell lymphoma cells (45).

LRG1, another upregulated protein in NHL serum, was indicated to be involved in various cancers (46–50). Aberrant expression of serum LRG1 was correlated with a number of colonic adenomas (50). Furthermore, the expression level of LRG1 was associated with the localization and tumor volume of colorectal carcinomas and had predictive value for the early diagnosis and prognosis of colorectal cancer combined with other serum markers (47, 51, 52). Accumulating evidence suggests that LRG1 could promote abnormal angiogenesis by modulating endothelial TGF-β signaling but plays a smaller role in normal blood vessel growth (53, 54). Aberrant neovascularization contributes to cancer, so LRG1 might be a potential target for regulating pathological angiogenesis (55). In hematological malignancies, LRG1 was elevated in the serum of pediatric acute lymphoblastic leukemia at the time of diagnosis (8). Moreover, the expression levels of serum LRG1 returned to normal in pediatric B-ALL patients achieving complete remission after induction therapy (56). LRG1 gene silencing promoted cell apoptosis by downregulating antiapoptotic proteins and upregulating proapoptotic proteins in AML KASUMI-1 cells (57).

To date, there has been no evidence available concerning the S100A8 and LRG1 proteins, and abnormal expression of serum S100A8 and LRG1 in pediatric NHL from published proteomics data. Here, our study found that serum levels of S100A8 and LRG1 in NHL group significantly increased compared to control group. ROC curves showed that the combination of S100A8 and LRG1 had higher diagnostic values in screening patients with NHL. These results indicate that S100A8 and LRG1 might be promising biomarkers for pediatric NHL. Hence, it is speculated that S100A8 and LRG1 may contribute to the occurrence and progression of pediatric NHL. However, this study was only a preliminary study with a relatively small sample size. Although, the current study was limited by the relative small sample size, our results were able to prove statistically significant differences between NHL and the controls. Therefore, in future trials, the series of DEPs should be further validated in large-scale samples. In addition, the children with NHL in our study were diagnosed at an advanced stage (III/IV), which may be related to the high heterogeneity and invasiveness of the malignant cells and poor expression ability of children. Further studies using early cases are guaranteed to verify the potential clinical value of identified DEPs.

## Conclusion

In conclusion, this is the first study using an iTRAQ-based proteomics approach to screen and identify serum DEPs of pediatric NHL, which could provide potential biomarkers for the early diagnosis, contributing to further understanding the pathogenesis of NHL and providing important experimental data for pediatric NHL. Among the large number of DEPs identified, serum S100A8 and LRG1 could serve as potential biomarkers for NHL diagnosis in the future. Further studies are needed to confirm and validate the DEPs on a large scale.

## Data Availability Statement

The raw data of mass spectrometry can be found in the iProx database (accession number: IPX0004164000). Further inquiries can be directed to the corresponding authors.

## Ethics Statement

This study followed the Declaration of Helsinki and was approved by the Institutional Ethics Committee of the Department of Medicine of the First Affiliated Hospital of Zhengzhou University. Informed consent was obtained from the parents or guardians pScience Foundation of Henan Province (212300410243rior to study initiation. All serum samples were discarded after clinical use. Written informed consent to participate in this study was provided by the participants’ legal guardian/next of kin.

## Author Contributions

Sample collection and data analysis were performed by LC and SY. LC performed the depletion of high-abundance proteins and protein quantification of serum. RY performed iTRAQ labeling and two-dimensional LC-MS/MS. SY performed ELISA validation and statistical analysis. YL and ZZ validated bioinformatics and statistical methods. The first draft of the manuscript was written by RY. All authors contributed to the article and approved the submitted version.

## Funding

This research was supported by funding from the Natural Science Foundation of Henan Province (212300410243), the Henan Provincial Science and Technology Research Project (212102310900) and the Henan Province Medical Science and Technology Tackling Program joint co-construction project (LHGJ20210056).

## Conflict of Interest

The authors declare that the research was conducted in the absence of any commercial or financial relationships that could be construed as a potential conflict of interest.

## Publisher’s Note

All claims expressed in this article are solely those of the authors and do not necessarily represent those of their affiliated organizations, or those of the publisher, the editors and the reviewers. Any product that may be evaluated in this article, or claim that may be made by its manufacturer, is not guaranteed or endorsed by the publisher.

## Supplementary Material

Additional File 1 Differentially expressed proteins identified in our study.

Additional File 2 Protein–protein interaction network analysis of differentially expressed proteins identified in B-NHL compared with the control.

Additional File 3 Protein–protein interaction network analysis of differentially expressed proteins identified in T-NHL compared with the control.
