# Supplementary figures and images for: iTRAQ-Based Proteomic Analysis Reveals Potential Serum Biomarkers for Pediatric Non-Hodgkin’s Lymphoma
Source: Front Oncol. 2022 Mar 17;12:848286. doi: 10.3389/fonc.2022.848286 (PMC8970600; doi:10.3389/fonc.2022.848286)

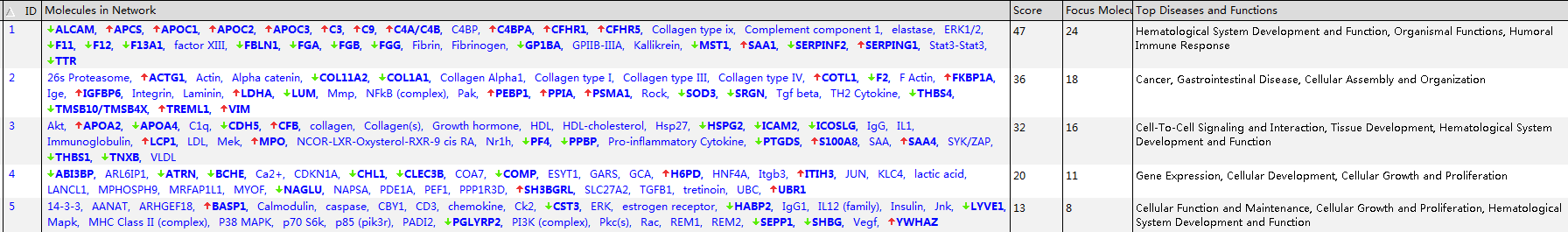

Supplement: Additional File 2 — Protein–protein interaction network analysis of differentially expressed proteins identified in B-NHL compared with the control. [file Image_1.png]

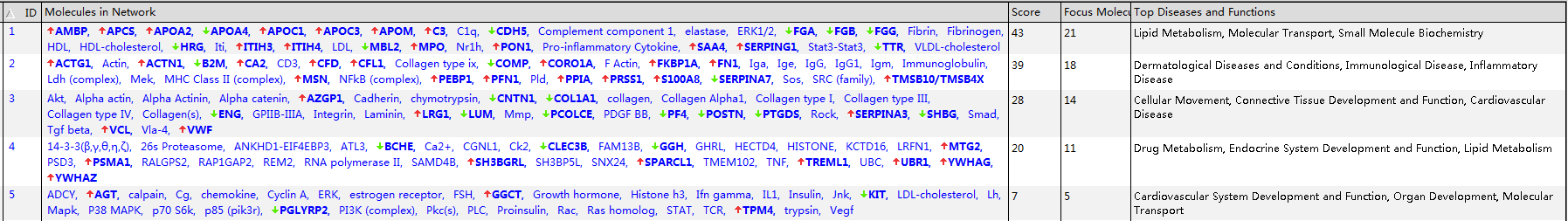

Supplement: Additional File 3 — Protein–protein interaction network analysis of differentially expressed proteins identified in T-NHL compared with the control. [file Image_2.png]
